# Are injured workers with higher rehabilitation service utilization less likely to be persistent opioid users? A cross-sectional study

**DOI:** 10.1186/s12913-019-3879-6

**Published:** 2019-01-14

**Authors:** Alyson K. Kwok, Nathan N. O’Hara, Andrew N. Pollak, Lyndsay M. O’Hara, Alexandra Herman, Christopher J. Welsh, Gerard P. Slobogean

**Affiliations:** 1 Glenrose Rehabilitation Hospital, Edmonton, AB Canada; 2 University of Maryland School of Medicine, Baltimore, MD USA

**Keywords:** Rehabilitation, Opioid use, Workers’ compensation, Injury, Service utilization

## Abstract

**Background:**

Given its role in treating musculoskeletal conditions, rehabilitation medicine may be an important factor in decreasing the use of opioids among injured workers. The primary objective was to determine if increased utilization of rehabilitation services was associated with decreased persistent opioid use among workers’ compensation claimants. The secondary objective was to determine the combined association of rehabilitation service utilization and persistent opioid use with days of work lost due to injury.

**Methods:**

Using Chesapeake Employers’ Insurance Company claims data from 2008 to 2016, claimants with at least one filled opioid prescription within 90 days of injury were eligible for inclusion. The primary outcome was persistent opioid use, defined as at least one filled opioid prescription more than 90 days from injury. The secondary outcome was days lost due to injury. The primary variable of interest, rehabilitation service utilization, was quantified based on the number of rehabilitation service claims and grouped into five levels (no utilization, and four quartiles - low, medium, high, very high).

**Results:**

Of the 9596 claimants included, 29% were persistent opioid users. Compared to claimants that did not utilize rehabilitation services, patients with very high rehabilitation utilization were nearly three times more likely (OR: 2.71, 95% CI: 2.28–3.23, *p* < 0.001) to be persistent opioid users and claimants with low and medium levels of rehabilitation utilization were less likely to be persistent opioid users (low OR: 0.20, 95%: 0.14–0.27, *p* < 0.001) (medium OR: 0.26, 95% CI: 0.21–0.32, *p* < 0.001). Compared to claimants that did not utilize rehabilitation services, very high rehabilitation utilization was associated with a 27% increase in days lost due to the injury (95% CI: 21.9–32.3, *p* < 0.001), while low (− 16.4, 95% CI: -21.3 – -11.5, *p* < 0.001) and medium (− 11.5, 95% CI: -21.6 - -13.8, *p* < 0.001) levels of rehabilitation utilization were associated with a decrease in days lost due to injury, adjusting for persistent opioid use.

**Conclusion:**

Our analysis of insurance claims data revealed that low to moderate levels of rehabilitation was associated with reduced persistent opioid use and days lost to injury. Very high rehabilitation utilization was associated with increased persistent opioid use and increased time from work.

## Background

Opioid drug use in the United States is alarmingly high, with at least 214 million prescriptions dispensed every year since 2006 [1]. Along with the increase in opioid use, over 11.5 million Americans reported misuse of prescription opioids and more than 14,000 people died from a prescription opioid overdose in 2016 [2, 3]. The United States Department of Health and Human Services has recently declared a public health emergency to address the opioid crisis [4]. Opioid prescriptions for injured workers have demonstrated a similar rate of increase since 2003 [5].

According to the United States Bureau of Labor Statistics, there were approximately 2.9 million nonfatal workplace injuries and illnesses in private industry in 2016, which occurred at a rate of 2.9 cases per 100 full-time equivalents [6]. The treatment of a workplace injury is often multi-modal and typically includes physical and occupational therapy as forms of rehabilitation medicine. Depending on the injury, an individual may receive multiple treatments during the course of his or her recovery. Rehabilitation practitioners have a variety of tools and techniques available intended to alleviate pain, improve function, and promote recovery. Exercise therapy, in particular, is well supported in the treatment of low back pain [7, 8], and osteoarthritis of the knee and hip [8–11]. Given its role in treating musculoskeletal conditions, rehabilitation medicine may be an important factor in decreasing the use of opioids among injured workers.

Opioid therapy for the treatment of a workplace injury has been estimated to more than double the likelihood of the claim exceeding $100,000 [12]. Several studies have demonstrated the increased risk of long-term disability and opioid dependence with opioid therapy after the acute phase on injury [13–17]. Dosing guidelines, such as those implemented by Washington State, have been successful in reducing chronic opioid use in the state’s workers’ compensation program [18]. However, additional strategies are likely required to fully curb this public health crisis.

The primary objective of this study was to determine if an increased utilization of rehabilitation services was associated with decreased persistent opioid use in workers’ compensation claimants. The secondary objective of the study was to determine the combined association of rehabilitation service utilization and persistent opioid use with days of work lost due to injury. We hypothesized that higher rehabilitation service utilization would be associated with lower persistent opioid use [19]. Our secondary hypothesis was that higher rehabilitation utilization would be associated with fewer days of work lost due to injury when controlling for persistent opioid use.

## Methods

### Study population

We examined insurance claims data from Chesapeake Employers Insurance Company (CEIC) from January 1, 2008 to December 31, 2016. CEIC is the largest writer of workers’ compensation insurance in the State of Maryland, providing coverage to approximately 266,000 workers in 2016. This study was approved by the University of Maryland Institutional Review Board. Informed consent was waived for the study as the dataset was de-identified. Data were extracted for each year of the study. During the study period, there were 100,357 unique claims included in the dataset. For patients with unique injury claims in multiple years of the study, only the first claimed injury was included in our analysis. Patients that died as a result of the claimed injury were excluded. All included patients claimed at least one opioid prescription.

### Outcome measures

The primary outcome for this study was persistent opioid use, defined as at least one filled opioid prescription paid for by the workers’ compensation claim more than 90-days from the date of injury. To be categorized as a persistent opioid user, the participant must have also filled at least one opioid prescription in the first 90-days post-injury. The 90-days from injury time frame is consistent with recent publications on persistent opioid use [20, 21]. The secondary outcome was days lost due to injury, as recorded on the workers’ compensation claim.

### Rehabilitation service utilization

Rehabilitation service utilization was the primary variable of interest and was quantified based on the number of rehabilitation procedures (*Current Procedural Terminology (CPT)* codes 97001–97799) included for a single individual in a workplace injury claim. The most common rehabilitation codes used in the claims data were 97110 – therapeutic procedure (36% of rehab claims), 97010 – application of a modality (19% of rehab claims), 97140 – rehabilitation therapeutic procedure (11% of rehab claims), and 97112 – therapeutic procedure (5% of rehab claims). Claimants were categorized by their level of rehabilitation service utilization. The categories for this variable included “no utilization”, plus the four quartile ranges. “Low utilization” ranging from 1 to 7 claims, “medium utilization” ranging from 8 to 37 claims, “high utilization” ranging from 38 to 147 claims, and “very high utilization” for those with more than 147 claims. The categorization of rehabilitation utilization in the sensitivity analyses was similarly based on the distribution quartiles.

### Included covariates

Sociodemographic, clinical, and employment variables were extracted from the insurance claims data. Demographic characteristics included age and sex. *International Classification of Diseases*, *9th Revision* (ICD-9) codes were used to define the mechanism of injury and categorized into high energy, low energy, or other/unspecified. The claimant’s disability status associated with the injury was categorized based on the most severe level of the claim as adjudicated by the Maryland Workers’ Compensation Commission and included five levels; medical-only claims, permanent total disability, permanent partial disability, total compensation, and temporary partial compensation. Surgical procedures were determined based on claimed CPT codes. Additional joint pain diagnoses were also recorded based on ICD-9 and *International Classification of Diseases, 10th Revision* (ICD-10) codes. The employment type was categorized using Standard Occupational Classifications and the employer was coded as the State or a private company [22]. The number of years with the current employer was extracted from the claims data.

### Statistical methods

Characteristics of the study population were summarized with counts and proportions and compared across the five levels of rehabilitation utilization using Chi-squared tests. Logistic regression was used for our primary analysis to test the association between rehabilitation utilization and persistent opioid use. The model was adjusted for age, mechanism of injury, disability status, type of occupation, years with current employer, receiving surgical treatment for the injury, and a concomitant diagnosis of chronic joint pain. For the secondary analysis, the outcome variable (days lost due to injury) was generalized logarithm transformed due to a highly skewed distribution. Multivariable linear regression was used to model the association between days lost due to injury and level of rehabilitation utilization, controlling for persistent opioid use, the interaction between rehabilitation utilization and persistent opioid use, and adjusting for patient age, sex, mechanism of injury, disability status, type of occupation, employer type, years with current employer, receiving surgical treatment for the injury, and a concomitant diagnosis of chronic joint pain.

An additional set of sensitivity analyses were performed to assess the effect of rehabilitation utilization within 90 days of injury and, specifically, active rehabilitation procedures within 90 days of injury. CPT codes were classified as either active or passive procedures based on the methods described by Fritz et al. [23]. The primary and secondary models were replicated using these two unique exposure variables. The correlation between rehabilitation utilization within 90-days of injury and rehabilitation utilization after 90 days from injury was tested using Spearman’s rank correlation.

All statistical analyses were performed using JMP Pro Version 13 (SAS Institute, Cary, NC).

## Results

**Fig. 1:**
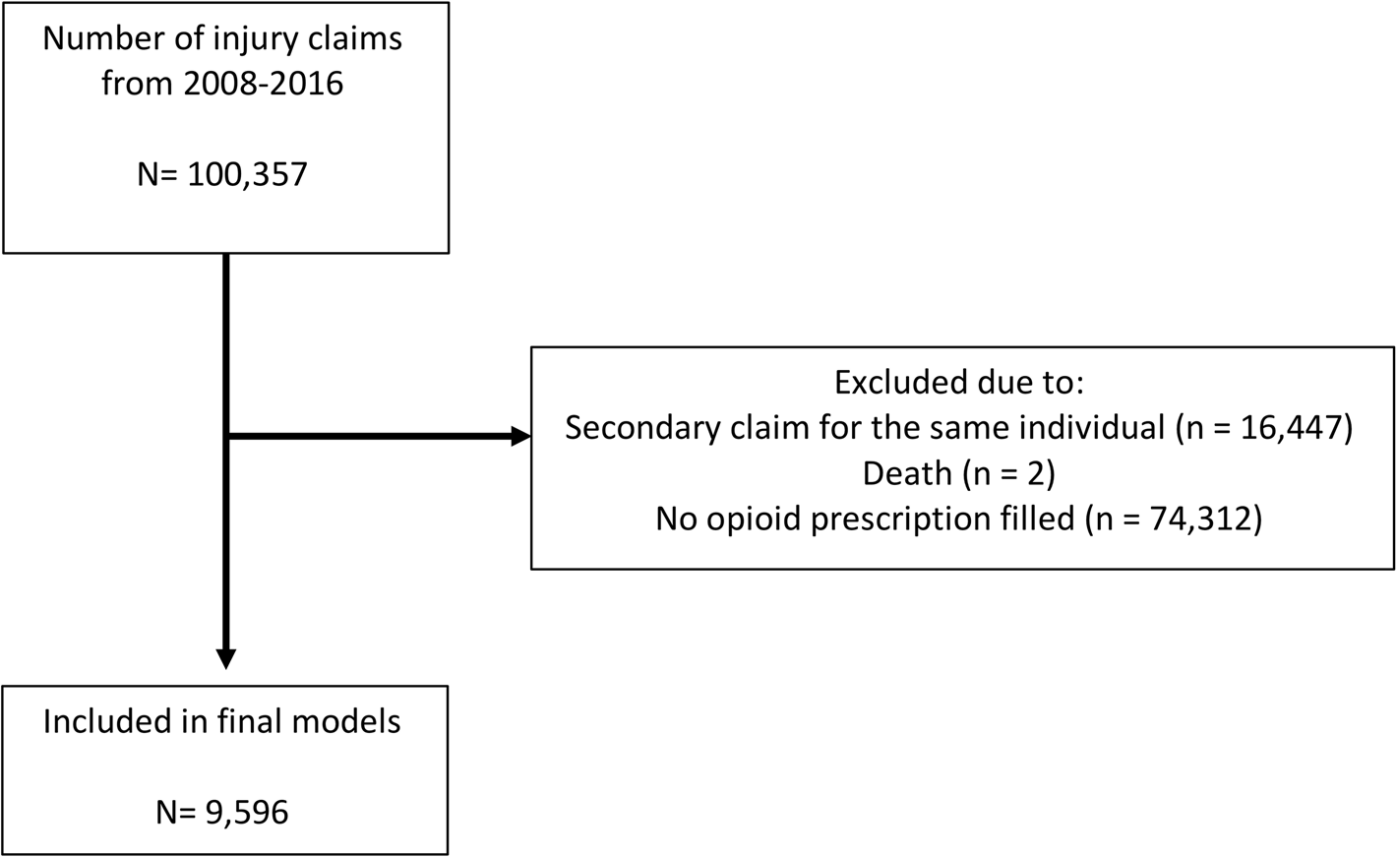
Patient flow diagram

Between January 1, 2008 and December 31, 2016, 100,357 unique claims were registered. Claims pertaining to a second injury during the study period led to the exclusion of 16,477 claimed injuries. Two subjects died as a result of their workplace injury and were excluded. An additional 74,266 individuals were excluded from the data for not having a single opioid prescription filled as part of their insurance claim. Nine thousand five hundred ninety-six employees were included for analysis (Fig. 1).

The injured patient characteristics are described in Table 1. Claimants were most commonly between the ages 30–49 (*n* = 4822, 50.3%) and male (*n* = 6217, 65.1%). Over 60% sustained a high energy injury (*n* = 5827). Operators and technicians were the most common occupation (*n* = 3111, 32.4%), followed by service workers (*n* = 2680, 27.9%). Most were employed by private companies (*n* = 6549, 68.2%). Fourteen-percent of the sample received surgical treatment for their injury (*n* = 1325) and 37.7% had a concomitant diagnosis of chronic joint pain (*n* = 3616).

**Table 1:** Patient characteristics (*n* = 9596)

	Levels of Rehabilitation Utilization										*P*-Value
	Very High		High		Medium		Low		No		
	*N* = 2397		*N* = 2398		*N* = 2314		*N* = 1070		*N* = 1417		
	N	%	N	%	N	%	N	%	N	%	
Age											
30 or less	299	12.5%	436	18.2%	559	24.2%	291	27.2%	280	19.8%	<0.001
30–49	1293	53.9%	1255	52.3%	1112	48.1%	511	47.8%	651	45.9%	
50 or more	805	33.6%	707	29.5%	643	27.8%	268	25.0%	486	34.3%	
Sex											
Male	1560	65.2	1493	62.5	1598	69.6	648	61.2	918	65.1	<0.001
Female	833	34.8	895	37.5	699	30.4	411	38.8	493	34.9	
Mechanism of Injury											
High Energy	1519	63.4	1465	61.1	1337	57.8	602	56.3	904	63.8	<0.001
Low Energy	576	24.0	635	26.5	641	27.7	281	26.3	345	24.3	
Other/Unspecified	302	12.6	298	12.4	336	14.5	187	17.5	168	11.9	
Disability Status											
Permanent Total Disability	9	0.4	1	0.0	0	0.0	0	0.0	1	0.1	<0.001
Permanent Partial Disability	1586	66.2	1208	50.4	362	15.6	31	2.9	525	37.1	
Temporary Total Compensation	583	24.3	716	29.9	794	34.3	186	17.4	425	30.0	
Temporary Partial Compensation	56	2.3	32	1.3	20	0.9	2	0.2	18	1.3	
Medical-Only	163	6.8	440	18.4	1138	49.2	851	79.5	448	31.6	
Type of Occupation											
Operatives & Technicians	841	35.1	724	30.2	787	34.0	309	28.9	450	31.8	<0.001
Service Workers	685	28.6	749	31.2	561	24.2	278	26.0	407	28.7	
Laborers & Helpers	418	17.4	427	17.8	436	18.8	176	16.4	236	16.7	
Professionals	197	8.2	190	7.9	208	9.0	131	12.2	138	9.7	
Office Workers	180	7.5	215	9.0	202	8.7	120	11.2	132	9.3	
Sales Workers	62	2.6	79	3.0	104	4.5	47	4.4	44	3.1	
Not recorded	14	0.6	20	0.8	16	0.7	9	0.8	10	0.7	
Type of Employer											
Private Employer	1576	65.7	1529	63.8	1676	72.4	797	74.5	971	68.5	<0.001
State Employer	821	34.3	869	36.2	638	27.6	273	25.5	446	31.5	
Years with Current Employer Prior to Claim											
Less than 2	759	31.7	840	35.0	866	37.4	441	41.2	479	33.8	<0.001
2–10	692	28.9	662	27.6	691	29.9	304	28.4	416	29.4	
More than 10	458	19.1	461	19.2	389	16.8	150	14.0	222	15.7	
Not recorded	488	20.4	435	18.1	368	15.9	175	16.4	300	21.2	
Surgically Treated Injury											
Yes	232	9.7	345	14.4	601	26.0	147	13.7	0	0.0	<0.001
No	2165	90.3	2053	85.6	1713	74.0	923	86.3	1417	100.0	
Diagnosis of Chronic Joint Pain											
Yes	1618	67.5	1256	52.4	637	27.5	105	9.8	0	0.0	<0.001
No	779	32.5	1142	47.6	1677	72.5	935	90.2	1417	100.0	

Two thousand seven hundred fifty-one (28.6%) claimants filled an opioid prescription more than 90 days from their date of injury. Patients with very high rehabilitation utilization (more than 147 claims) were nearly three times (OR: 2.71, 95% CI: 2.28–3.23, *p* < 0.001) more likely to fill a subsequent opioid prescription more than 90 days from injury than patients with no rehabilitation service claims, in our adjusted analysis (Table 2). Claimants with low (range: 1–7 claims) and medium (range: 8–37 claims) levels of rehabilitation utilization were less likely to fill a subsequent opioid prescription more than 90 days from injury (low OR: 0.20, 95%: 0.14–0.27, p < 0.001) (medium OR: 0.26, 95% CI: 0.21–0.32, *p* < 0.001) when compared to injured workers that did not have a single rehabilitation service claim. High levels of rehabilitation utilization (range: 38–147) were found to be moderately protective against persistent opioid use, in our adjusted analysis (OR: 0.80, 95% CI: 0.67–0.94, *p* = 0.01).

**Table 2:** The association between persistent opioid use and level of rehabilitation utilization

Rehabilitation Utilization	Unadjusted			Adjusted		
	OR	95% CI	*P*-Value	OR	95% CI	*P*-Value
Very high	4.01	3.48–4.63	<0.001	2.71	2.28–3.23	<0.001
High	1.03	0.89–1.19	0.70	0.80	0.67–0.94	0.01
Medium	0.23	0.19–0.28	<0.001	0.26	0.21–0.32	<0.001
Low	0.12	0.09–0.17	<0.001	0.20	0.14–0.27	<0.001
No	Reference (1.00)			Reference (1.00)		
Model Fit						
*R*^2^	0.18			0.21		

*OR* odds ratio, *CI* confidence interval

Over 40% of the cohort (*n* = 4082) did not lose any work days due to their injury. The median days of work missed due to injury was 17 days (IQR: 0–136). Very high rehabilitation utilization was associated with a 27.1% increase in days lost due to the injury (95% CI: 21.9–32.3, *p* < 0.001) compared to patients that did not use rehabilitation services (Table 3). Low (− 16.4, 95% CI: -21.3 – -11.5, *p* < 0.001) and medium (− 11.5, 95% CI: -15.8 - -7.3, p < 0.001), levels of rehabilitation utilization were associated with a decrease in days lost due to injury compared to patients who did not use rehabilitation services. There was no difference in days lost due to injury between patients with high levels of rehabilitation utilization and patients who did not use any rehabilitation services. Persistent opioid use was associated with increased days lost due to injury by 17.3% (95% CI: 14.6–20.0, *p* < 0.001), in the adjusted analysis.

**Table 3:** The association between days lost due to injury and level of rehabilitation utilization, persistent opioid use

Rehabilitation Utilization	Unadjusted			Adjusted		
	Percent Change	95% CI	*P*-Value	Percent Change	95% CI	*P*-Value
Very high	61.3	53.5–69.0	<0.001	27.1	21.9–32.3	<0.001
High	20.4	13.6–27.2	<0.001	−2.6	−7.1 – 1.8	0.25
Medium	−16.6	−23.2 - -10.1	<0.001	−11.5	−15.8 -7.3	<0.001
Low	− 64.8	−72.5 - -57.2	<0.001	−16.4	−21.3 - -11.5	<0.001
No	Reference (0.00)			Reference (0.00)		
Persistent Opioid Use						
Yes	73.1	62.8–83.4	<0.001	33.6	27.2–39.9	<0.001
No	Reference (0.00)			Reference (0.00)		
Model Fit						
Adjusted *R*^2^	0.22			0.71		

In the first sensitivity analysis, very high rehabilitation utilization within 90 days of injury was associated with a 21% increase in the odds of persistent opioid use (very high OR: 1.21, 95% CI: 1.07–1.38, *p* < 0.01) compared to study participants with no rehabilitation claims (Table 4). However, there was no association between other levels of rehabilitation utilization within 90 days of injury and persistent opioid use. Similarly, there was no association between the utilization of active rehabilitation services and persistent opioid use. The number of rehabilitation sessions within 90 days of injury has a low correlation with the number of rehabilitation sessions that occurred more than 90 days from injury (ρ = 0.44, *p* < 0.001) [24].

**Table 4:** The association between persistent opioid use and level of rehabilitation utilization (total procedures and active procedures) within 90 days of injury

Rehabilitation Utilization	Unadjusted			Adjusted		
	OR	95% CI	*P*-Value	OR	95% CI	*P*-Value
Total sessions within 90 days of injury						
Very high	2.08	1.86–2.33	<0.001	1.21	1.07–1.38	<0.01
High	1.44	1.28–1.62	<0.001	1.01	0.89–1.15	0.87
Medium	0.91	0.54–1.52	0.71	0.65	0.38–1.13	0.13
Low	1.22	0.92–1.62	0.16	1.05	0.77–1.42	0.76
No	Reference (1.00)			Reference (1.00)		
Active rehabilitation sessions within 90 days of injury						
Very high	1.84	1.65–2.06	<0.001	1.09	0.96–1.23	0.18
High	1.34	1.18–1.53	<0.001	0.99	0.86–1.14	0.84
Medium	1.09	0.79–1.50	0.58	0.76	0.54–1.06	0.10
Low	1.32	1.07–1.64	0.01	1.05	0.83–1.32	0.70
No	Reference (1.00)			Reference (1.00)		

Note: Active and passive rehabilitation procedures were determined based on Current Procedure Terminology codes, as described by Fritz et al

The second sensitivity analysis observed a similar association between very high rehabilitation utilization (total and active) with an increase in the days lost due to injury (Table 5). Less rehabilitation utilization was associated with fewer days lost due to injury. In contrast to the primary model, lower levels of rehabilitation utilization did not reduce the number of days lost due to injury compared to study participants that did not use rehabilitation services.

**Table 5:** The association between days lost due to injury and level of rehabilitation utilization within 90 days of injury (total and active procedures), persistent opioid use

	Unadjusted			Adjusted		
	Percent Change	95% CI	*P*-Value	Percent Change	95% CI	*P*-Value
Total sessions within 90 days of injury						
Rehabilitation Utilization						
Very high	58.6	53.1–64.2	<0.001	28.4	24.4–32.5	<0.001
High	25.9	20.6–31.2	<0.001	12.1	8.3–15.9	<0.001
Medium	27.1	6.7–47.6	0.01	14.0	0.0–28.0	0.05
Low	11.6	0.0–24.0	0.07	9.1	0.00–17.8	0.04
No	Reference (0.00)			Reference (0.00)		
Persistent Opioid Use						
Yes	61.6	54.6–68.7	<0.001	21.8	16.7–26.9	<0.001
No	Reference (0.00)			Reference (0.00)		
Active rehabilitation sessions within 90 days of injury						
Rehabilitation Utilization						
Very high	51.7	46.2–57.2	<0.001	25.1	21.1–29.1	<0.001
High	29.4	23.5–35.4	<0.001	15.1	10.9–19.2	<0.001
Medium	24.2	10.2–38.3	<0.01	8.1	−0.1 – 17.8	0.10
Low	19.2	9.3–29.2	<0.01	13.3	6.4–20.2	<0.001
No	Reference (0.00)			Reference (0.00)		
Persistent Opioid Use						
Yes	63.3	56.6–70.0	<0.001	24.1	19.2–28.9	<0.001
No	Reference (0.00)			Reference (0.00)		

Note: Active and passive rehabilitation procedures were determined based on Current Procedure Terminology codes, as described by Fritz et al

## Discussion

Among workers’ compensation claimants in the State of Maryland, we observed a strong association between rehabilitation utilization and persistent opioid use even when adjusted for age, mechanism of injury, disability status, type of occupation, years with current employer, receipt of surgical treatment for the injury, and a concomitant diagnosis of chronic joint pain. Low and medium levels of rehabilitation utilization, in particular, were associated with reduced persistent opioid use compared to claimants who did not utilize rehabilitation services. Similarly, 1 to 37 rehabilitation service claims were associated with fewer days lost due to injury compared to claimants who did not utilize rehabilitation services, in the adjusted analysis. The protective effects of rehabilitation against persistent opioid use and days lost due to injury did not extend to claimants with high and very high levels of rehabilitation utilization, which was counter to our hypothesis. Claimants with very high levels of rehabilitation utilization (more than 147 service claims) were more likely to be persistent opioids users and to have more work days lost due to injury.

The sensitivity analyses suggest that the level of early rehabilitation utilization had less of an effect on persistent than the overall level of rehabilitation utilization. In contrast to the main secondary analysis, low and medium levels of rehabilitation utilization within 90 days of injury did not reduce the number of days lost due to injury when compared to study participants that did not attend a single rehabilitation session. However, this result may be confounded by nuances to the types and severity of injury that was not available in the data. The findings also suggest a modest benefit from active rehabilitation compared to passive rehabilitation within 90 days of injury in reducing the likelihood of persistent opioid use and reducing the number of days lost due to injury.

Our findings suggest that there may be an optimum level of rehabilitation utilization, a concept supported by prior work-disability duration research. Wasiak et al. found that shorter chiropractic care duration was associated with shorter work-disability duration [25]. Additionally, a 2015 study found fewer physical medicine and rehabilitation services and fewer passive services in the first 8 weeks after workplace injury was associated with shorter work-disability duration [26]. Our analysis of claims data found the benefits associated with rehabilitation extended to a threshold of approximately 40 treatments over the duration of their claim. Treatment extended beyond this point, where recovery is likely beginning to plateau, could lead the injured worker to seek or rely on other treatments such as opioids.

Persistent opioid use was associated with very high rehabilitation utilization (147 or more rehabilitation service claims), as well as increased days lost to injury. It is possible that persistent opioid use may impact the recovery process, however, causation cannot be established in this study design. Previous studies have observed an association between early opioid prescription and delayed recovery [27], and early opioid prescriptions as a risk factor for work disability greater than or equal to 6 months after the incident [28]. Similarly, a 2015 study found that early reimbursement for opioids predicted prolonged claim duration [17]. Our study provides additional data to infer the impact of rehabilitation service utilization in combination with persistent opioid use on days lost due to injury.

We utilized a workers’ compensation claims database with a robust capture of baseline characteristics, injury, and treatment details spanning 9 years. Despite these strengths, some limitations must be considered in the interpretation of the findings. The definition of persistent opioid use is based on prescriptions filled, however, we are unable to determine if the medication was actually consumed by the study participant. The categorization of rehabilitation use was based on the quartiles observed in the data and may not be consistent with the frequency of rehabilitation use in other workers’ compensation populations. A precise measure of injury severity was not available in the data, and while the adjusted models included the adjudicated disability status of the claim, this may not be correlated with the severity of injury. A concomitant diagnosis of previous alcohol or substance abuse as well as anxiety and mood disorders have been previously associated with persistent opioid use but were infrequently reported in the claims data and therefore not included in our analysis [21]. Underlying patient traits, such as patient motivation and involvement, were also not measured in these data. Likewise, potential secondary gain from continued opioid or higher rehabilitation utilization cannot be determined. Additionally, the details of the rehabilitation received are not available, such as the provision of multi-disciplinary care, the quality of the rehabilitation received, or the presence of a return to work program. There is evidence that a multidisciplinary pain management program may be effective in reducing opioid use for patients with chronic, non-cancer pain [29]. Working for an employer with a return to work program has also been associated with fewer claim days [17].

Further studies, in the form of well-designed randomized controlled trials, are needed to determine the effect of the number of physical therapy sessions on days lost due to injury and persistent opioid use. The findings of this study support a hypothesis of diminishing marginal gains from more than 40 rehabilitation service claims and negative implications for days lost due to injury and persistent opioid use at levels of rehabilitation service utilization exceeding 147 claims. It is possible that capping the number of rehabilitation sessions available to injured workers may improve the patient’s activation during the rehabilitation stage of recovery. This policy may also encourage the utilization of other therapeutic modalities in combination or as a substitute for physical therapy. Absent of strong evidence to support an upper limit to effective rehabilitation utilization, many healthcare providers and payers are impugned to discontinue physical therapy treatment. However, if supported, this simple policy change may lead to significant benefit and reduced cost for the rehabilitation of injured workers.

## Conclusion

Our analysis of insurance claims data revealed that low to moderate levels of rehabilitation was associated with reduced persistent opioid use and days lost to injury compared to no rehabilitation. Very high rehabilitation utilization was associated with increased persistent opioid use and increased time away from work.

## Abbreviations

CEIC: Chesapeake Employers Insurance Company
CI: Confidence Interval
CPT: Current Procedural Terminology
ICD: International Classification of Diseases
OR: Odd Ratio
